# Seaweed as Basis of Eco-Sustainable Plastic Materials: Focus on Alginate

**DOI:** 10.3390/polym16121662

**Published:** 2024-06-12

**Authors:** Ismael Santana, Manuel Felix, Carlos Bengoechea

**Affiliations:** Escuela Politécnica Superior, Universidad de Sevilla, Calle Virgen de África, 7, 41011 Sevilla, Spain; isantana@us.es (I.S.); mfelix@us.es (M.F.)

**Keywords:** alginate, hydrogel, bioplastic, marine resources, biopolymer, processing, seaweed

## Abstract

Seaweed, a diverse and abundant marine resource, holds promise as a renewable feedstock for bioplastics due to its polysaccharide-rich composition. This review explores different methods for extracting and processing seaweed polysaccharides, focusing on the production of alginate plastic materials. Seaweed emerges as a promising solution, due to its abundance, minimal environmental impact, and diverse industrial applications, such as feed and food, plant and soil nutrition, nutraceutical hydrocolloids, personal care, and bioplastics. Various manufacturing techniques, such as solvent casting, injection moulding, and extrusion, are discussed for producing seaweed-based bioplastics. Alginate, obtained mainly from brown seaweed, is particularly known for its gel-forming properties and presents versatile applications in many sectors (food, pharmaceutical, agriculture). This review further examines the current state of the bioplastics market, highlighting the growing demand for sustainable alternatives to conventional plastics. The integration of seaweed-derived bioplastics into mainstream markets presents opportunities for reducing plastic pollution and promoting sustainability in material production.

## 1. Introduction

The term algae refers to a large phylogenetic unit that includes cyanobacteria, microalgae and macroalgae, and seaweed [1]. Seaweeds are macroscopic multicellular photosynthetic marine organisms [2,3] that usually grow in intertidal zones commonly between 30 and 40 m from the tidal channel but can reach depths of up to 180 m, mainly on solid substrates such as rocks, shells, and other materials. They can also be found in shallow coastal waters or estuaries [4]. Seaweeds have been employed since ancient times mainly in Asian countries [5,6], but nowadays, due to their availability and large diversity, seaweeds are considered globally as a novel source of bioactive components, such as peptides, amino acids, proteins, and polysaccharides. Thus, seaweeds have been used in a wide range of applications [7], such as human nutrition (43.77%) [8], animal feed (3.86%) [9], bioplastic production (5.34%) [10], and nutraceuticals (1.93%) [11]. As most of the species have no applications for human consumption, and can even act as invasive species when landing in different environments [12], they can be used as raw materials for the development of biodegradable materials [13], in line with the aim of a circular economy [14].

### 1.1. Macroalgae Composition

**Figure 1:**
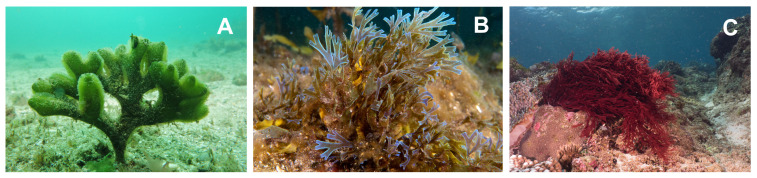
Images of some green, brown, and red seaweeds: (**A**) *Codium* sp.; (**B**) *Dictyota* sp.; and (**C**) *Gracilaria* sp. (**A**,**B**) images by John Turnbull and (**C**) by Rickard Zerpe, available at flickr.com.

There are about 10,000 species of macroalgae estimated, classified into three main groups based on their pigmentation (Figure 1), i.e., green (Chlorophyceae), brown (Phaeophyceae), and red (Rhodophyceae) [15,16]. The composition of seaweeds varies depending on several factors such as the group and species to which they belong, the environmental conditions (e.g., temperature), the season, or the harvest location [17]. The approximate composition of different algae is presented in Table 1.

**Figure 2:**
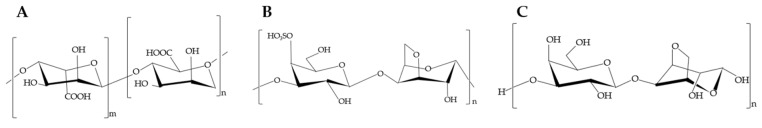
Chemical structure of (**A**) alginate, (**B**) carrageenan, and (**C**) agar.

Carbohydrates are the major components of most seaweeds, mainly found in the form of polysaccharides, typically ranging from 4% to 76% of their dry weight [18,19]. The polysaccharides from macroalgae of commercial importance are alginate (30,000 tons/year, 12 USD/kg), carrageenan (60,000 tons/year, 10.4 USD/kg), and agar (10,600 tons/year, 18 USD/kg) [10,20,21]. Their chemical structure can be observed in Figure 2. Alginate is a linear polysaccharide mainly extracted from brown algae, which can be found in its acid form, as alginic acid or as a salt-forming part of the cell wall [22]. Its structure is composed of two hexuronic monomers, α-1-glucuronic acid (G) and β-d-mannuronic acid (M), linked by (1-4)-glycoside linkages [23]. Carrageenans are linear polysaccharides which can be found in the cell wall of mainly red algae, formed by alternating chains of sulphated and non-sulphated galactose chains linked by glycosidic bonds [24]. Agar is also extracted mainly from red algae and consists of a mixture of polysaccharides composed of agaropectin and agarose [25]. The most widespread use of alginate, carrageenan, and agar extracted from seaweed is as a gelling and thickening agent in the food industry [26].

The protein fraction found in seaweeds is also very variable depending on the seaweed type, the season, and place of collection [27,28]. Brown seaweeds are the ones with the lowest protein content, with a maximum of ~20% (dry basis). On the other hand, green and especially red seaweeds have, in general, a higher protein content, comparable with vegetables such as soybeans or other legumes (~40%, dry basis) [29,30]. Thus, the nori variety is a red seaweed with up to 32.2% protein content [31], while green seaweeds such as some from the Ulva family contain up to 38.16% [32]. These proteins have an amino acid composition like that of legumes, glutamic acid and aspartic acid being the most abundant amino acids [33]. On the other hand, free amino acid fractions are composed mainly of alanine, aminobutyric acid, citrulline, hydroxyproline, ornithine, and taurine, the amount depending on the species of seaweed [24].

The lipid content is generally low in seaweeds compared to animal sources, with a ~1–5% (dry basis) of lipids, its fatty acid profile depending on the species and environmental conditions [33]. Regarding this, a higher percentage of fatty acids has been observed in winter or spring than in summer and in cold-water species compared to tropical ones [34,35]. The lipids of marine algae are mainly composed of polyunsaturated fatty acids, the content of which increases in species from cold climates [27,36].

**Table 1:** Proximate composition of different seaweeds.

Pigmentation	Seaweed	Lipids	Carbohydrates	Protein	Ashes	Moisture	Reference
Green	*Undaria pinnatifida*	0.9 ± 0.1	32.4	23.8 ± 0.6	30.62 ± 0.25	11.77 ± 0.01	[27]
Green	*Codium tormentosum*	3.6 ± 0.2	32.8	18.8 ± 0.1	35.99 ± 0.48	9.0 ± 0.2	[27]
Red	*Gracilaria gracilis*	0.60 ± 0.01	46.6	20.2 ± 0.6	24.8 ± 0.03	7.99 ± 0.02	[27]
Red	*Grateloupia turuturu*	2.2 ± 0.1	43.2	22.5 ± 0.3	20.52 ± 0.01	11.68 ± 0.05	[27]
Red	*Crassiphycus corneus*	1.74 ± 0.05– 1.93 ± 0.03	24.02 ± 2.23– 23.55 ± 3.01	22.93 ± 0.16– 21.27 ± 0.21	26.11 ± 0.06– 34.16 ± 0.06	5.24 ± 0.12– 4.30 ± 0.06	[37]
Green	*Ulva fasciata*	2.76 ± 0.34– 2.37 ± 0.09	42.24 ± 0.70– 40.91 ± 0.28	17.97 ± 0.15– 11.42 ± 0.16	16.51 ± 0.85– 20.89 ± 0.76	7.28 ± 0.34– 10.29 ± 0.33	[37]
Brown	*Sargassum vulgare*	4.11 ± 0.03– 4.02 ± 0.19	28.30 ± 0.32– 39.07 ± 1.34	14.02 ± 0.24– 10.32 ± 0.04	36.79 ± 0.76– 30.09 ± 0.33	6.76 ± 0.06– 4.53 ± 0.08	[37]
Brown	*Alaria esculenta*	1.30 ± 0.05	-	9.11 ± 0.57	24.56 ± 0.56	5.39 ± 0.05	[38]
Brown	*Laminaria digitata*	1.13 ± 0.05	-	5.31 ± 0.34	24.43 ± 0.03	6.81 ± 0.06	[38]
Brown	*Rugulopteryx okamurae*	11.63 ± 0.22	38.87 ± 0.40	9.93 ± 0.16	18.47 ± 0.35	13.48 ± 0.26	[39]
Brown	*Dictyota dichotoma*	4.70 ± 0.10	11.02 ± 0.09	4.32 ± 0.12	-	-	[40]

### 1.2. Macroalgae-Based Alternatives for Environmentally Friendly Packaging

The issue of plastic pollution has been lingering for years, observing an increasing trend. Worldwide, plastic is being generated at 400 Mt per year, of which 90.4% is fossil-based, and only 9% of plastics are recycled. Most of the plastic produced (i.e., ~80%) ends up being discarded [41]. Depending on their size, plastic waste in nature can be classified as follows: macroplastics (>25 mm), mesoplastics (5–25 mm), microplastics (<5 mm), and nanoplastics (<1 μm) [42]. It is challenging to separate plastic waste for recycling, leading to uncontrolled incineration or accumulation in nature. Due to the long time required for their degradation (500–1000 years), most plastic waste eventually ends up in oceans, where 8 to 10 Mt is found every year, accounting for 80% of all marine pollution [41,43,44,45]. Additionally, the long life cycle of conventional plastics requires increasing storage space, causing not only soil infertility but also significant issues with odours and potential diseases where they are deposited [46]. Taking all these data into account, it is expected that by 2030, an unsustainable limit of plastic pollution accumulation will be reached, estimated at around 53 Mt per year [47,48]. Although the transition to higher circularity has increased significantly in the last few years, still around one-quarter of plastic waste is sent to landfills in Europe (7.6 Mt in 2022). The most common use of conventional plastics is for packaging (39%) due to their low weight, good protective function, and low production cost. The biggest drawback is that its life cycle is extremely higher than that of packaged goods, leading to a fast pace in the generation of waste. In 2022, it was estimated that 57.3% of plastic waste came from packaging, followed by building and construction (7.1%) and automotive applications (5%) [41].

Macroalgae are made up of hydrocolloids, which are hydrophilic macromolecules that exhibit great solubility or swellability when immersed in water. The viscosity and the ability to form 3D networks of polysaccharides found in seaweed (i.e., alginate, carrageenan, agar) play a crucial role in the development of bioplastics [49,50]. A bioplastic is defined as a plastic material that is either biobased, biodegradable, or features both properties [51]. Seaweeds have already shown potential as a source of raw materials for the development of bioplastics [10,52]. There are strong reasons for employing bioplastics based on macroalgae as packaging materials: firstly, the inherent hydrocolloids within macroalgae could serve as fundamental constituents for innovative packaging materials entirely derived from macroalgal biomass [53]. Unlike synthetic polymer processes, which often require extensive extraction and reassembly, natural polymers from macroalgae can be liberated from cell walls with minimal chemical alteration; moreover, there is an assumption that these materials may exhibit comparable degradation rates to raw macroalgal biomass, especially evidenced by their rapid decomposition in polar solvents [54]; furthermore, the production and processing of macroalgal biomass into packaging material demand substantially less energy and resources compared to synthetic polymer counterparts. This translates into reduced costs associated with biomass and renewable energy consumption, presenting a more sustainable alternative [53]; lastly, the choice of using natural polymers refined from algae, as the main ingredient of the filler, can impart beneficial properties (e.g., antioxidant, antimicrobial) to the resulting packaging material [55,56,57]. Although the use of pure natural polymers offers the prospect of high-quality products, their availability may compete with other crucial applications in food, cosmetics, and medicine. Moreover, it should also be highlighted that the scalability of these processes currently represents a significant challenge for the bioplastics industry. Thus, studies have pointed out that the large-scale implementation of bioplastics is still far from being fully reached, as several obstacles delay their broad adoption [58]. Then, a proper solution is likely supposed to be found in a longer time than desired when bioplastics can be sold at similar or even lower prices than petroleum-based polymers. For this to happen, bioplastic technology should effectively improve as crude oil costs increase [58]. Furthermore, investment in bioplastics can yield long-term benefits in terms of sustainability and reduction in plastic pollution. In this sense, a recent report stated that the production of bioplastics from seaweed could be a cost-effective and efficient solution for the growing demand for sustainable plastic products [59].

### 1.3. Research Status

Seaweed-related patent publications have experienced a significant increase especially when compared to the ones focused on other marine genres. Most patents and scientific seaweed-based publications come from Asian countries (Japan, South Korea, and China) [60]. This is mainly due to the culture of these countries where seaweed has played a fundamental role in food, which is why most of their publications focus on the use of these algae in human nutrition [61]. On the other hand, seaweed-based patents and scientific publications in Western countries are moving away from human food to focus more on other utilities such as medicine, cosmetics, and biotechnology, diversifying the sectors of interest [62].

**Figure 3:**
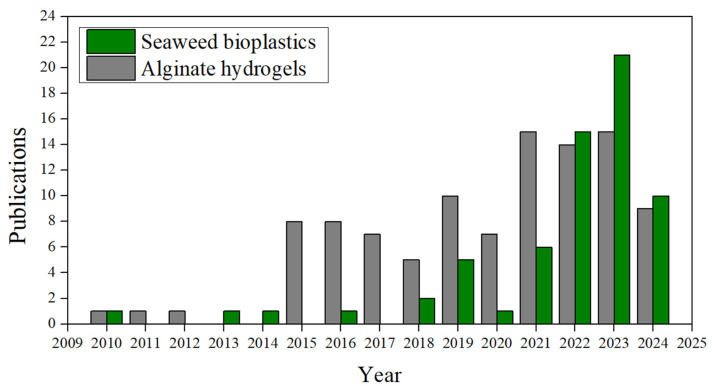
The number of publications per year from January 2010 to May 2024 using the search criteria “Seaweed bioplastic” (grey) and “Alginate” AND “hydrogel” and “seaweed” (green) on the Scopus website.

As the present review focuses on the feasibility of using seaweed as a source of raw materials to produce bioplastics, mainly for sustainable packaging applications, the research status was analysed through a search on the Scopus database with the search criterion “seaweed bioplastic”, where 54 results were obtained from 2010 to 21 May 2024 [63]. Following this, another search criterion was applied to “alginate” AND “hydrogel” AND “seaweed” to find more specific publications on hydrogels produced from alginate extracted from macroalgae, obtaining 95 results. Figure 3 shows the results of the number of publications per year of each criterion. A significant increase in publications is observed from 2019 to 2022, except for 2020, compared to previous years.

Focusing on the 64 scientific publications about “seaweed bioplastic”, 38 are articles, 15 reviews, and the rest are contributions to conferences and others. Although they are distributed in different subject areas, which may overlap, the categories with the highest number of contributions are “Environmental Sciences”, with 22 publications, “Chemical Engineering”, with 13, and “Chemistry” and “Materials Science”, with 11 and 8, respectively. As for the search criteria for alginate hydrogels within the same timeframe, 102 results were obtained, comprising 61 scientific articles, 19 book chapters, and 17 reviews. The remaining entries originated mainly from short surveys or conference papers. The most common subject area was “material science,” with 44 results, followed by “chemistry” with 31, “chemical engineering” with 28, and “engineering” with 23. There are fewer contributions to other areas, such as 15 for “Agricultural and biological sciences” or 7 results for “Environmental science”, among many others.

A growing trend in research can be observed in both topics (seaweed bioplastics and alginate hydrogels), especially since 2015. This growth is more pronounced in studies on alginate hydrogels extracted from seaweed, increasing from one in 2010 to eight in 2015, maintaining this general trend until late 2023. Regarding research on bioplastics derived from seaweed, the growth is more gradual over the years but shows a greater interest than that for alginate hydrogels from 2022 onwards. In the trend of both, a small dip in the number of publications is noticeable in 2020, likely influenced by the effects of the COVID-19 global pandemic.

The current state of research indicates that its valuable composition and wide availability in nature make macroalgae interesting for a wide variety of uses in industries, whether food, pharmaceutical, or materials, among others. In essence, the adoption of macroalgae for bioplastics presents potential benefits such as efficient production, enhanced biodegradability, minimized environmental impact, and versatility in material quality. Nonetheless, further research is imperative to comprehensively evaluate the environmental implications and material characteristics of macroalgae-based bioplastics. In this review, an analysis will be made of the different uses and composition of the several types of macroalgae, emphasizing the use of macroalgae in the development of bioplastics as sustainable materials and the advances in research in this regard in recent years.

### 1.4. Current State of Bioplastics Market

At the beginning of the 21st century, the consumption of conventional plastics was estimated to be 15 kg/year per person which, with the increase in the world population, has been increasing up to now [64]. Due to their low price, versatility, and durability, conventional plastics are the most used materials ordinarily, which implies an industrial demand of production of about 380–390 Mt, of which 50% are plastics of one single use [65].

The introduction of bioplastics in markets has historically been difficult due to several factors. One of them is the stability of the nearly 100 years of the conventional plastics industry, with well-defined production stages and means, stable economic models, and study of consumption. This is why one way to broaden the inclusion of bioplastics in the industry compared to conventional plastics is by obtaining materials that, by reducing or equalling costs and having similar mechanical properties, manage to reduce the environmental impact [66]. Around this idea of concern for the environment and the development of bioplastics, the distinction between compostable, degradable, and biodegradable plastics was introduced [67]. This, together with the policies introduced by several countries at the beginning of the 21st century that supported this type of material, increased interest in development in the field of bioplastics [68].

According to the data from the Plastics Europe report, the global production of biobased plastics (including bio-attributed plastics) increased from 1.4 Mt in 2020 to 2.3 Mt in 2022, which accounts for 0.57% of the total plastics production. In Europe, this number increases to 1% of the total plastics production [69], and it is expected to increase in the next few years. At the global level, Europe produces 27% of bioplastics (with Germany contributing 50.9% of this share) second only to China, which produces 33%. The main application of bioplastics today is for packaging (almost 40%), followed by building and construction materials, the automotive industry, and others [70].

According to forecasts by the European Bioplastic Association, from 2022 to 2023, maintaining a utilization rate of 82–83% every year in bioplastic production capacities, the actual global production increased from 1.83 Mt to 2.18 Mt. These production capacities of bioplastics and biobased and biodegradable plastics are expected to reach 2.67 Mt this year and nearly double by 2025, reaching a global production of approximately 4.83 Mt, with an upward trend in the following years [71].

As around 70% of the surface of the earth is covered with water, there is a great availability of seaweed that can be used as raw material for the development of bioplastics [72]. The fact that the cultivation of seaweed for its different uses does not compete with other crops or livestock in terms of the need for land or drinking water implies a great advantage for the sector when it comes to taking it into account as a material for the food, cosmetics, pharmaceutical, or biotechnological industry, among others [73], leaving seaweed in an advantageous position compared to other raw materials. However, it is crucial to consider the ecological implications of massive seaweed harvesting. Excessive collection can alter marine ecosystems, affecting biodiversity and ecological functions. Habitat reduction for various marine species and changes in ecosystem structure are among the potential negative effects, which stresses the importance of implementing sustainable harvesting practices to mitigate these impacts and promote the conservation of the marine environment [74].

## 2. Extraction and Application of Seaweed Components

### 2.1. Biorefinery

There are many components of seaweed that can be used in industry. Among them, the one found in the highest proportion by weight and that is the most used in industry is phycocolloids, which are polysaccharides extracted from seaweed, mainly alginate, carrageenans, and agar [75]. The extraction of practically any compound begins with a waster wash to eliminate salt and impurities from the seaweed, drying, either by freeze-drying or spray-drying, and its subsequent grinding to increase the contact surface of the algae with the solvents. There are several extraction processes for these polysaccharides, but only the most used ones will be explained. According to the World Bank Group, agar, alginate, and carrageenan have an annual production of 10,600, 30,000 and 60,000 Mt/year, respectively, with a corresponding retail price of 18, 12, and 10.4 USD/kg. This implies a market value of USD 191, 339, and 626 million per year, respectively [21].

- Alginate

The extraction of alginate from seaweed is based on the precipitation process, either of the calcium salt or of the alginic acid. Industrially, the process through the formation of calcium alginate is preferred due to the ease of separation using industrial filters [76]. Alginate extraction generally begins with a treatment with 2% formaldehyde to remove pigments, followed by an acid wash (e.g., HCl, H_2_SO_4_) to break down the cell wall and facilitate the extraction of the alginate from dried and milled seaweed. The resulting extract is heated and treated with Na_2_CO_3_ to obtain insoluble sodium alginate. The sodium salt of alginate is precipitated with ethanol, separated by centrifugation, and washed with methanol and acetone [75].

- Carrageenan

Carrageenan, mainly obtained from red seaweed, is extracted in high yield (higher than 40%) by alkaline treatment [77]. To conduct this, the dry and grinded seaweed is soaked into an alkaline solution. The resulting carrageenan gel is separated from the rest of the solution by filtration. The application of heating and ultrasound may increase the extraction yield, as shown in the results obtained by Martin del Campo et al. [77] for the extraction of carrageenan from *Chondracantus canaliculatus* red seaweed or by Webber et al. from *Kappaphycus alvarezii* [78].

- Agar

Agar extraction is the simplest process compared to other phycocolloids. Starting from the dried and ground seaweed, it is immersed in hot water for a few hours, and then the agar dissolved from the solid residue is filtered. The agar is finally allowed to gel and dried by spray-drying or freeze-drying to obtain a powder. The application of the ultrasound of an alkaline treatment can increase the extraction yield, while the application increases the purity of the agar obtained, although it typically reduces the extraction yield [79].

### 2.2. Industrial Applications of Seaweed

*A* series of cascading processes are used in the conversion of seaweed biomass in a sustainable and profitable biorefinery so that the most valuable products are used in pharmaceuticals, and the least valuable are biofuels [80]. Alginate, mainly obtained from brown algae, is one of the most used polysaccharides due to its gelling properties, although they depend greatly on its monomer sequence and composition [81]. The properties of alginate gels are mainly given by its molecular structure, and it has great use both in the food industry and outside it, either as a gelling agent, fertilizer, or drug release matrix [82,83]. Carrageenans are also widely used in industry, especially the food industry, thanks to their thickening, gelling, and stabilizing properties. The number of sulphated groups connected to the galactose monomer determines the type of carrageenan (kappa, iota, or lambda, among others) and its rheological properties [84]. In nature, mixtures of the several types of carrageenans are generally obtained, but some algae mainly produce certain types compared to others, such as red seaweed *Kappaphycus alvarezii*, which mainly produces the kappa monomer [85]. Its main use is as an additive in the food industry (mainly lambda and kappa) [86] or in the medical industry thanks to its antiviral, anticoagulant, and antithrombin capacity [87]. Agar is a polysaccharide chain composed of agarose to a greater extent and agaropectin [88]. The most used seaweeds in the industry for agar extraction are Gracilaria, cultivated mostly in Chile and Indonesia and with suitable properties for the food industry thanks to its emulsifying, stabilizing, and thickening properties, and Gelidium, used in the pharmaceutical industry for its bacteriological activity [89].

Some of the applications of these seaweed components are summarized below, with special emphasis on bioplastics.

- Pharmaceutics

The pharmaceutical sector requires a higher level of evidence of effectiveness than the rest of the applications, as they should be demonstrated clinically before health claims can be stated [90]. Their main attractive quality comes from the ease of the gel formation of polysaccharides present in seaweeds, which can be better used as drug delivery systems, gelation kinetics being an important role in their effectiveness. Other applications within the pharmaceutical sector include scaffolds, bone tissue engineering, and cell encapsulation [91].

- Food

The use of seaweed as food was initially observed in Asian countries such as Japan in the 4th century or China in the 6th century. Currently, about 90% of the seaweed in the industry is used as food, especially in East Asian countries, although this is spreading to a lesser extent to Western countries [26]. Seaweed contains a significant amount of some health-promoting components, such as ω-3 fatty acids, essential amino acids, vitamins, and dietary fibre that helps to ameliorate digestive health [92].

- Bioplastics

Due to the overall increase in the global population and, consequently, rising consumption, bioplastics emerge as a viable alternative for certain applications, such as food packaging. The term “bioplastic” refers to plastics produced from renewable or biodegradable sources [45,93]. Bioplastics used in the industry exhibit several key characteristics that make them appealing as sustainable alternatives to conventional plastics. These include biodegradability, renewability, biocompatibility, a diverse range of properties, and reduced carbon emissions during production [94]. In addition to their environmental benefits, bioplastics used in the industry must also possess suitable rheological properties to meet processing requirements. These properties include melt flow behaviour, viscosity, and elasticity, which impact the manufacturability and performance of bioplastic products. Ensuring appropriate rheological characteristics is essential for successful integration into industrial processes [95].

A very promising use of seaweed is as a feedstock for producing bioplastics thanks to its high polysaccharide content and variety. Polysaccharides such as cellulose, alginate, carrageenan, and agar are the main components of seaweed used in the production of bioplastics, although in some cases, the complete crude extract of the seaweed is also used [10,52]. It is necessary to carry out a study on the mechanical properties, such as their tensile strength, Young’s modulus, or elongation at break, of the products developed from seaweed components or extract to assess their success in replacing the common plastics used.

Bioplastics from brown algae *Laminaria japonica* and *Sargassum natans* were produced from the whole seaweed by Doh et al. (2020) [96]. Generally, when working with crude extracts, a plasticizer such as glycerol is usually used to obtain bioplastics with adequate mechanical properties. When considering seaweed extracts, the use of alginate or carrageenan as a basis for bioplastics is somehow restricted due to their high water vapor permeability (WVP) and low water resistance [97,98]. However, there are different strategies to overcome that limitation. Alginates can be blended with starches to obtain biodegradable plastic films with low gas permeability and improved mechanical properties [91]. Also, the addition of nanoparticles or clays can help to improve the properties of alginate-based materials, promoting the extension of the shelf life of a variety of products. For example, alginate-based films are proven to protect meat products from the loss of moisture or colour loss, gas transfer, or prevent microbial contamination [99]. Alginate-based bioplastics, especially when used with plasticizers like glycerol, represent a promising alternative in terms of sustainability and mechanical properties. However, for applications requiring high flexibility and elongation, the advantages offered by traditional plastics like PET are apparent. The future development of bioplastics could focus on improving elongation at break without compromising other mechanical properties. Some authors, such as Gao et al., previously produced bioplastics with alginate as a base, with and without plasticizer, achieving promising mechanical properties. Without plasticizer, they obtained a Young’s modulus of 2417 ± 197 MPa, a tensile strength of 38.5 ± 4.3 MPa, and an elongation at break of 3 ± 1%. When 10% glycerol was used as a plasticizer, these values varied, yielding a Young’s modulus of 934 ± 97 MPa, a tensile strength of 20.8 ± 2.3 MPa, and an elongation at break of 12 ± 3% [100]. More traditional plastics, such as polyethylene terephthalate (PET), have Young’s modulus values of 2016 MPa, a tensile strength of 50.45 MPa [101,102], and an elongation at break of 230% [102]. Further research is then required to find an adequate replacement for traditional plastics.

When considering carrageenan for food containment applications, several physical and chemical techniques have been studied, such as blending carrageenans with hydrophobic compounds, the use of nanomaterials as reinforcement agents, or layered composites, which have significantly enhanced carrageenan film properties [98]. Agar also has hygroscopic properties and is inert, which makes it a great candidate to produce transparent biofilms with heat sealability when mixed conveniently with a plasticizer [103]. The drawbacks it may present (i.e., brittleness, poor elasticity and thermal stability, medium gas barrier properties, high water sensitivity, high WVP) could be alleviated with novel formulations including blends with other biopolymers, oils, nanoparticles, or antimicrobial and/or antioxidant agents [104]. In this sense, agar-based films have shown similar Young’s modulus values to starch-based films and coatings, although below that of most commercial polymers [105]. Moreover, in most cases, the cost of producing packaging from seaweed is still much higher than the petroleum-derived counterparts, and there is a great amount of work to be conducted in this aspect to extend their application [104].

- Fertilizer

Seaweed has long been used as fertilizer, whether as mulch, compost, or more. Currently, fertilizers are obtained from complete macroalgae or their by-products through different techniques such as grinding, hydrolysis, or acid extraction [106,107]. The success of using seaweed as fertilizers lies mainly in its composition, since it has essential nutrients for plants such as nitrogen, magnesium, phosphorus, calcium, or traces of metals such as zinc or iron [107]. This translates into a wide variety of benefits for plants, depending on when and how it is used, improving the general growth of the plant and protecting it against pathogenic microorganisms and different environmental conditions [108].

- Biofuel

Although it is not widely used, it is possible to use biomass from seaweed to produce biogas. The main problem that this industry presents is the cost of collection from coasts and farms, process performance, and the need for desalination plants for subsequent anaerobic digestion [109,110]. This is why it presents great economic disadvantages. On the other hand, these projects are supported by anti-pollution laws, and there are initiatives to use algae as biofuel in countries such as Brazil and South Korea [111].

## 3. The Processing of Seaweed for the Manufacture of Materials

Seaweed has been considered as a raw material for the new generation of biologically derived plastic products. Plastics based on seaweed have the benefit of a lower environmental impact in terms of no land use being required for their cultivation [112]. However, more research is still needed to upscale and commercialize these novel bioplastics. To obtain seaweed with a significant percentage of the desired biopolymer, it is usual to harvest specific seaweed cultures under controlled conditions that promote the development of seaweed polysaccharides optimally [113]. After harvest, seaweed is typically dried, washed, and grinded to remove impurities [114]. Then, a seaweed flour is obtained that can be further processed to obtain the desired plastic product.

### 3.1. Processing Techniques for Alginate Bioplastics

#### 3.1.1. Solvent Casting

One of the most used methods to produce alginate bioplastic films is wet formation or solvent casting. The first stage of the process consists of dissolving the polymer in a suitable solvent, which may require the adjustment of pH, temperature, or the addition of other compounds. After homogenizing the solution, it is poured into a suitable mould or onto a Teflon-coated plate, allowed to dry, and, finally, the film obtained is separated [115,116]. This method has been used to produce bioplastics from various seaweeds, such as by S. Hii et al. [117] by using the agar extracted from the red seaweed *Glacilaria salicornia* or from alginate extracted from *Sargassum* sp. by Kanagesan et al. [118]. Ayala et al. performed a Life Cycle Assessment for the pilot-scale production of a seaweed bioplastic through the casting method. They determined that 9.058 L of the alginate-rich crude extract from *Saccharina latissima* brown seaweed was required to produce a kilogram of bioplastic, the use of glycerol as the plasticizer being the greatest contributor to the global warming impact [112].

#### 3.1.2. Injection Moulding

Injection moulding is a widely used method to produce plastic materials, although not so much for alginate bioplastics [119,120,121,122]. The process consists of the softening of the polymeric raw material by heating and its introduction through pressure inside a mould. Normally, it is necessary to premix the polymers and plasticizers using a mixer. The process can be divided into four stages: (i) in the first, the material is heated in a barrel to the temperature that allows for the material to flow; (ii) in the second phase, injection takes place, where a piston applies a certain pressure that pushes the sample into the cavities of the mould at the moulding temperature; (iii) then, a holding stage takes place once the blend is in the mould cavities, where temperature and pressure are kept to an optimum level so that crosslinking permits us to obtain a stable plastic material; and (iv) finally, the mould is cooled to be able to manipulate and extract the sample [123]. This technique is highly versatile as products of different shapes could be obtained. Not many research studies include the injection moulding of seaweed biopolymers. Bioplastics based on *Rugulopteryx okamurae* brown seaweed were produced using this method by Santana et al. [39], and composites based on polylactic acid (PLA) together and a by-product of the extraction of alginate from seaweed were processed through injection moulding by Bulota and Budtova [124].

#### 3.1.3. Extrusion

Extrusion is a widely utilized method for polymer processing. It involves a metallic cylinder, capable of heating, housing either one or two internal screws, depending on whether it is a single- or twin-screw extruder configuration, which transport the polymer from a hopper to the exiting die. The screw generates pressure and shear forces throughout the cylinder, which is heated through frictional heating and external heat input [125].

In a single-screw extruder, the friction between the material and the screw contributes to the heating of the material, along with external heating. Solvent evaporation may occur upon the material exiting the extruder when the extruded materials contain volatile solvents. The premixing of components in a mixer before extrusion may be necessary in some cases, depending on the material and extruder design. Conversely, in twin-screw extrusion, material adhesion to the screws is reduced due to the collaborative action of both screws, although this can vary depending on the design and operating conditions [126].

Also, regarding the treatment of macroalgae and their by-products, such as alginate, to produce bioplastics through extrusion, two different processing models are usually used: wet and dry [53]. The difference between wet and dry extrusion lies in the moisture and temperature conditions during the process. Wet extrusion involves coating or encapsulating high-moisture products using an alginate-based material that gels in the presence of divalent cations. This process is carried out at high moisture and low temperature, followed by a crosslinking step to strengthen the coating. In contrast, dry extrusion is performed at very high temperatures and low moisture levels, like the conventional extrusion of thermoplastic polymers. Here, macroalgal biomass or derived polymers are blended with conventional thermoplastic polymers, but the resulting product is non-recyclable and compromises biodegradability [53]. Extrusion has been used to produce composites of alginate and PLA [127] or lignocellulosic derivatives [128], even applying four recycling steps in the latter case, without significantly affecting its properties.

### 3.2. Manufacture of Alginate Gels

One of the main advantages of alginate compared to other polysaccharides extracted from algae is its ability to form gels despite variations in temperature. Alginate gels could be dried and then rehydrated conveniently when needed so that these dried gels could be seen as novel bioplastic materials. Some authors have studied the effect of freeze-drying and rehydrating alginate gels stating that the release behaviour of rehydrated gels was not affected when in the form of nanoparticles [129].

**Figure 4:**
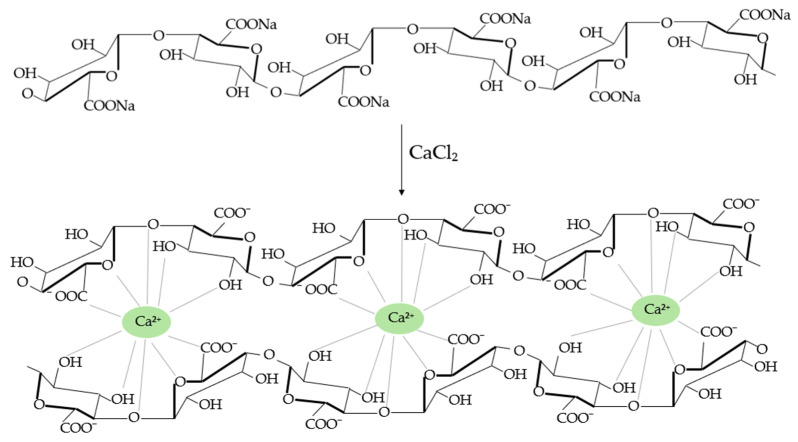
“Egg box” structure of calcium alginate gels.

Alginate gelation can occur through acid deposition or cation binding [83]. The properties of the formed gels depend on the molecular mass, which is directly related to the number and arrangement of M and G monomers that form the alginate chain and may vary among different algal species. The primary and most prevalent method for producing alginate gels involves bivalent cations, typically Ca^2+^. Depending on the method employed, the conversion ratio to calcium alginate, the source of Ca^2+^ ions, and the molecular structure of the alginate itself, the properties of the resulting gels can be varied [130]. This variation arises from gelation occurring through the binding of Ca^2+^ ions to the carboxyl groups of two guluronic acid monomers, thus forming a three-dimensional structure known as an “egg box”, shown in Figure 4, which imparts significant stability to the macromolecule [131,132].

Although the stability of the gel and pore size usually increases with the amount of G blocks forming alginate, the relationship between gel stability and pore size should be considered, which is not straightforward. While more stable gels may exhibit larger pores due to reduced contraction, pore size and distribution are also influenced by other factors, including alginate and calcium concentration, as well as the gelation method employed. This can result in a range of pore sizes, typically between 5 and 200 nm, allowing for permeability to small molecules such as insulin or glucose [83,133].

There are different methods for producing alginate gels industrially. Different reviews and book chapters deal with processing techniques to produce these alginate gels [83,134], this specific subject being out of the scope of the present review.

#### Applications of Alginate Gels in Industry

Alginate gels are commonly employed in the form of gel particles to encapsulate food, pharmaceutical, biomedical, and agriculture materials. Moreover, within the food industry, most uses focus on the thickening, water retention, or formation of protective films [83]. In the pharmaceutical industry, it is used as a carrier and deliverer of drugs, and they are ideal for producing nanoparticles due to their biodegradability, biocompatibility, availability, and low cost [82]. The use of alginate gels in food packaging is particularly advantageous due to their biodegradability, biocompatibility, and excellent barrier properties against gases and oils, which help extend the shelf life of food products, as mentioned before. Additionally, alginate gels can form flexible and resilient films suitable for various shapes and sizes of foods and can incorporate antimicrobial compounds to prevent the growth of microorganisms [135]. An example in the industry is the company Notpla, which creates biodegradable and edible packaging such as “Ooho” made from seaweed and plants [136].

## 4. Conclusions

The abundance and diversity of seaweeds make them a promising source of renewable and sustainable bioplastic materials. This study has explored methods for extracting and processing algal polysaccharides, focusing on alginate, carrageenan, and agar, and has examined various manufacturing techniques for producing bioplastics, especially from alginate. The extraction of seaweed typically begins with harvesting, drying, washing, and milling, followed by specific polysaccharide extraction processes, such as alginate gel formation through calcium ion binding. The application of methods such as solvent casting, extrusion, and injection provides versatile options for bioplastic manufacturing from algal polysaccharides.

Alginate, with its unique gelling properties, shows significant potential in a variety of industries, including pharmaceutical, agricultural, and food, highlighting its ability to encapsulate and release drugs and its use as a thickening and water retention agent. Additionally, agar and carrageenan also offer valuable properties, such as emulsifying and stabilizing in the food industry and the formation of robust films in packaging applications.

The bioplastics market is experiencing notable growth, driven by increasing concerns about plastic pollution and the search for sustainable alternatives. While bioplastics represent a relatively small fraction of the global plastics market, their share is growing, especially in packaging applications. This growth is expected to continue in the coming years, particularly with the backing of government policies and growing environmental awareness. The use of seaweed as a raw material for bioplastics presents numerous environmental and economic benefits. The abundant availability of seaweed offers a renewable source with low impact, as its cultivation does not require land use, as conventional agricultural crops. Furthermore, the ability of algae to absorb CO_2_ and nutrients from surrounding waters can contribute to mitigating ocean acidification and improving water quality. As the research and development of algae-based bioplastics continue to advance, it is crucial to address several challenges, such as optimizing extraction and manufacturing processes, improving mechanical properties, and evaluating the biodegradability and environmental toxicity of bioplastics production. Additionally, collaboration between industry, academia, and policymakers is essential to foster the adoption and commercialization of algae-based bioplastics.

In summary, seaweeds represent a valuable source of sustainable raw materials for bioplastics production with significant potential to reduce dependence on conventional plastics and mitigate associated environmental impacts. The successful integration of seaweed-based bioplastics into global supply chains offers a unique opportunity to promote sustainability in materials manufacturing and address the current challenges of plastic pollution on a global scale.

## Data Availability

Data are contained within the article.
